# The influence of sodium on pathophysiology of multiple sclerosis

**DOI:** 10.1007/s10072-016-2802-8

**Published:** 2017-01-11

**Authors:** Jacek Zostawa, Jowita Adamczyk, Paweł Sowa, Monika Adamczyk-Sowa

**Affiliations:** 1 Department of Neurology in Zabrze, Medical University of Silesia, ul. 3-go Maja 13-15, 41-800 Zabrze, Poland; 2 Department of Otorhinolaryngology and Oncological Laryngology, Medical University of Silesia, ul. C. Skłodowskiej 10, 41-800 Zabrze, Poland

**Keywords:** Sodium chloride intake, Multiple sclerosis, Environmental factors in MS, VGSCs

## Abstract

Multiple sclerosis (MS) is a chronic, inflammatory, autoimmune disease of the central nervous system, and is an important cause of disability in young adults. In genetically susceptible individuals, several environmental factors may play a partial role in the pathogenesis of MS. Some studies suggests that high-salt diet (>5 g/day) may contribute to the MS and other autoimmune disease development through the induction of pathogenic Th17 cells and pro-inflammatory cytokines in both humans and mice. However, the precise mechanisms of pro-inflammatory effect of sodium chloride intake are not yet explained. The purpose of this review was to discuss the present state of knowledge on the potential role of environmental and dietary factors, particularly sodium chloride on the development and course of MS.

## Introduction

Multiple sclerosis (MS) is a chronic, inflammatory, autoimmune disease of the central nervous system (CNS), and is a major cause of disability in young adults [1]. In genetically susceptible individuals, environmental factors play a significant partial role in the pathogenesis of MS [2]. Numerous studies examined the influence of environmental factors, such as Epstein–Barr infection [3–5], vitamin D levels [6–11], smoking [12, 13], obesity [14], sunlight exposure [15, 16], and geographic variation, on MS [17, 18].

## Methodology

All quotable references were searched using Pubmed and Google scholar between March and May 2016. References that allowed free access to full text by the Medical University of Silesia were retrieved and read. The oldest publications were retrieved from the Medical University of Silesia Library. One reviewer performed literature searches and two other researchers independently screened the articles. All disagreements were discussed and resolved by the authors or in consultation with other experts. Information used in our review was evaluated using evidence-based medicine. Inclusion criteria for the articles were: original papers, systematic reviews, systematic summaries, and meta-analysis. We excluded publications written in foreign languages, such as Spanish, German, or Russian. Keywords used for literature searches were as follows: “multiple sclerosis”, “MS”, “sodium”, “sodium chloride”, “autoimmunity”, “diet”, and “VGSCs”. All keywords were in accordance with the MeSH terms. To identify the appropriate publications, we searched Pubmed and Google scholar using combinations of keywords in the following order: “MS and diet”, “MS and salt intake”, “MS and environmental factors,” and “MS and VGSCs”. After reading titles and abstracts, some articles from each combination of keywords were excluded. Most of the excluded articles were in a foreign language. The comprehensive literature search identified 907 articles that were relevant for our review. Titles, abstracts, or full articles were reviewed to determine whether each search result matched our selection criteria. We also reviewed the references of the selected original papers and review articles found by our search for additional papers relevant to our review. Only high-quality publications from the last decades were included. In addition, 306 articles were retrieved from the Medical University of Silesia Library. Of these 306 articles, we eliminated those that were too general in scope for our review.

## Environmental factors influencing MS

Although some studies confirmed the link between a previous infection with Epstein–Barr virus (EBV) and the development of multiple sclerosis [3–5, 19–21], the involvement of EBV in the etiology of MS is unclear. However, certain viral infections likely increase susceptibility to MS [22]. Sero-epidemiological studies have demonstrated that almost 100% of adult MS patients are infected with EBV [19]. Late childhood infection of EBV is proposed to be the serious risk factor for the disease. Moreover, there is a strong EBV-specific CD8+ response in the blood of MS patients in the beginning of the disease and the intensity decreases in the course of the illness [2–5, 19, 20].

Low serum vitamin D levels are currently one of the most studied environmental factors influencing the development of MS. It has been shown that intake of food rich in vitamin D significantly prevents the development of MS or reduce activity of the disease [6–9, 16]. Nevertheless, some authors did not report a protective role of vitamin D supplementation for the development of MS [9, 10].

Smoking is another possible factor for the development of MS or might be responsible for worst prognosis of course of the disease [13, 23]. Furthermore, smoking cessation improved the prognosis in patients with MS. Ramanujam et al. confirmed that the time to conversion to secondary progressive MS (SPMS) decreases by 4.7% for each additional year of smoking after the diagnosis in patients with RRMS (acceleration factor 1.047; 95% CI 1.023–1.072; *P*  <  0.01) [13].

Obesity is a probable susceptibility factor for MS and several other autoimmune diseases [24], but the relationship between increased body mass index (BMI) and disease activity has not been fully explained. There is no doubt that obesity increases levels of pro-inflammatory cytokines and is associated with low-grade inflammatory state [25]. There are reports providing that RRMS activity is higher in obese and overweight patients than in patients with normal BMI undergoing IFN β treatment [14]. Moreover, Oliveira et al. reported a positive relationship between elevated BMI and disability in MS patients [26].

Ultraviolet radiation was proposed to be a significant environmental factor influencing prevalence of the disease [15]. Reduced risk of MS through exposure to sunlight is probably mediated not only by increased production of vitamin D in the skin, but also by the synthesis of anti-inflammatory factors, such as IL-10, TNF-α, and Treg cells [16]. Therefore, the latitude is nowadays considered to be related with prevalence of MS. The disease is less frequent near the equator and more frequent in northern countries [18, 27]. Exceptions to this trend, namely, Sardinia, where the prevalence of the disease is significantly higher [28], and northern Scandinavia, with markedly low prevalence [29], may be due to genetic and behavioral factors [18].

## Influence of diet on MS

It seems that diet might have a significant relationship with the inflammatory process of MS. Many studies have shown that diet plays the role in the pathogenesis of MS [17, 30–32]. Recent studies have provided the evidence for a protective role of polyunsaturated fatty acids on the risk of MS; however, there is no conclusive evidence for a beneficial role of polyunsaturated fatty acid supplementation in patients with MS. Hoare et al. demonstrated that the amount of omega-3 polyunsaturated fatty acids taken orally is inversely proportional to the risk of demyelination in the CNS [33]. Moreover, Khalili et al. found a strong correlation between oral intake of lipoic acid (1.2 mg/day) and decrease in the levels of pro-inflammatory cytokines, including INF-γ, ICAM-1, and anti-inflammatory cytokines, including TGF-β and IL-4, compared with placebo group [35]. On the other hand, Torkildsen et al. showed that consumption of omega-3 fatty acids used as monotherapy or in combination with interferon beta-1a had no beneficial effect on the disease compared to placebo [34]. Retinoic acid (RA), an active metabolite of vitamin A, revealed a strong immunosuppressive activity [36]. RA has been shown to modulate the balance between Th1/Th2 and Th17/Treg cells and B cell function, contributing to augmented tolerance and inhibited inflammatory response. It also contributes to enhanced tolerance and reduction of inflammatory effects [37]. Bitarafan et al. investigated the impact of vitamin A on disease progression in MS patients. The study evaluated the expanded disability status scale (EDSS) and MS functional composite (MSFC). The results showed that vitamin A improved MSFC in RRMS patients, but did not affect EDSS, relapse rate, or active brain lesions in MRI [38]. Ketogenic diet (high amount of fat, decreased protein content, and very low carbohydrates) was shown as potentially therapeutic in progressive forms of MS, which is especially relevant, because currently, there is no treatment for progressive forms of the disease [39, 40]. Kim et al. reported that ketogenic diet improved motor disability and cognitive impairment in mice with experimental autoimmune encephalomyelitis compared with mice on the standard diet. Furthermore, a ketogenic diet reversed structural brain lesions and reduced CNS inflammation and oxidative stress [41]. On the other hand, it was reported that creatine supplementation did not improve muscle capacity or habitual fatigue in MS individuals [42] or that restricted intake of animal fat (no more than 10–15 g/day) caused remission of the disease in patients with RRMS [43].

Polyphenols and carotenoids from vegetables, n-3 PUFA from fish, vitamins A, C, D, and E, thiol compounds, such as lipoic acid, and oligoelements, such as selenium and magnesium, have anti-oxidant properties [44, 45]. Th17 cells, which produce pro-inflammatory cytokines, are increased, whereas Treg cells are decreased in MS, and thus, the balance between Th17 and Treg cells is impaired in this disease. Vitamin A and its active metabolites (all-trans-retinoic acid and 9-*cis*-retinoic acid) modulate the imbalance of Th17 and Treg cells and might be beneficial to the prevention and treatment of MS [46]. Moreover, this vitamin was proposed to have a beneficial effect during interferon therapy and improved psychiatric outcomes for anti-inflammatory mechanisms [37].

## Sodium channels in MS

Voltage-gated sodium channels (VGSCs) are key mediators of action potential initiation and propagation in excitable cells [47–49]. Their expression has also been reported in cell types that are traditionally regarded as non-excitable, including glia, human vascular endothelial cells, human epidermal keratinocytes, and carcinoma cells, where their role is less clear [50–55]. Aberrant functional expression/activity of VGSCs has been identified as a major contributing factor in a number of human pathologies, including cardiac arrhythmia [55], epilepsy [56, 57, 58], pain [59, 60], periodic paralysis [61, 62], migraine [63], MS [64], and cancer [65]. VGSCs exist as heteromeric membrane-bound protein complexes that typically consist of a single pore-forming α subunit in association with one or more β subunits [66, 67].

The mammalian sodium channels include ten members (Nav1.1–Nav1.9 and Nax) encoded by genes SCN1A–SCN11A. While substantial homology exists between the isoforms, differences in amino acid sequence confer distinct voltage dependence, kinetic and pharmacological properties on each of the isotypes [68, 69]. Data concerning the location and function of each VGSCs subunit are included in Table 1.

**Table 1:** Voltage-gated sodium channels (VGSCs)

Protein	Human gene	Location	Function
(A) The α subunits			
Na_v_1.1	*SCN1A*	CNS, PNS, heart	CBH, dementia [70], Dravet syndrome [71], epilepsy [71, 72]
Na_v_1.2	*SCN2A*	CNS, PNS	CBH, dementia [70], epilepsy [73, 74], autism [74]
Na_v_1.3	*SCN3A*	CNS, PNS	Diabetes [75], neuropathic pain [76–78]
Na_v_1.4	*SCN4A*	Skeletal muscle, heart	Brugada syndrome [79], myotonia, periodic paralysis [80]
Na_v_1.5	*SCN5A*	Uninnervated skeletal muscle, heart, brain	Breast cancer [81, 82], arrhythmia [83], Brugada syndrome [84], angiogenic disorders [85]
Na_v_1.6	*SCN8A*	CNS, PNS, heart	Epilepsy [86], cervical cancer [87]
Na_v_1.7	*SCN9A*	PNS, neuroendocrine cells, sensory neurons	Angiogenic disorders [85], paroxysmal extreme pain disorder [88]
Na_v_1.8	*SCN10A*	Sensory neurons	Prostate cancer [89], cardiac arrhythmia [90], MS [90, 91]
Na_v_1.9	*SCN11A*	Sensory neurons	Congenital insensitivity [92], cold-aggravated pain [93]
Na_x_	*SCN6A, SCN7A*	Heart, uterus, skeletal muscle, astrocytes, DRG	Atopic dermatitis [94], hypertension [95]
(B) The β subunits			
β1	*SCN1B*	Heart, skeletal muscle, CNS, glia, PNS	Epilepsy [96], cardiac arrhythmia [97], cancer [98]
β1A(β1B)	*SCN1B*	Heart, skeletal muscle, adrenal gland, PNS	Epilepsy [96]
β2	*SCN2B*	CNS, PNS, heart, glia	Altered pain response [59], MS [99]
β3	*SCN3B*	CNS, adrenal gland, kidney, PNS	Cancer [98]
β4	*SCN4B*	Heart, skeletal muscle, CNS, PNS	Huntington’s disease [100]

*CNS* central nervous system, *MS* multiple sclerosis, *PNS* peripheral nervous system, *CBH* chronic brain hypoperfusion

**Fig. 1:**
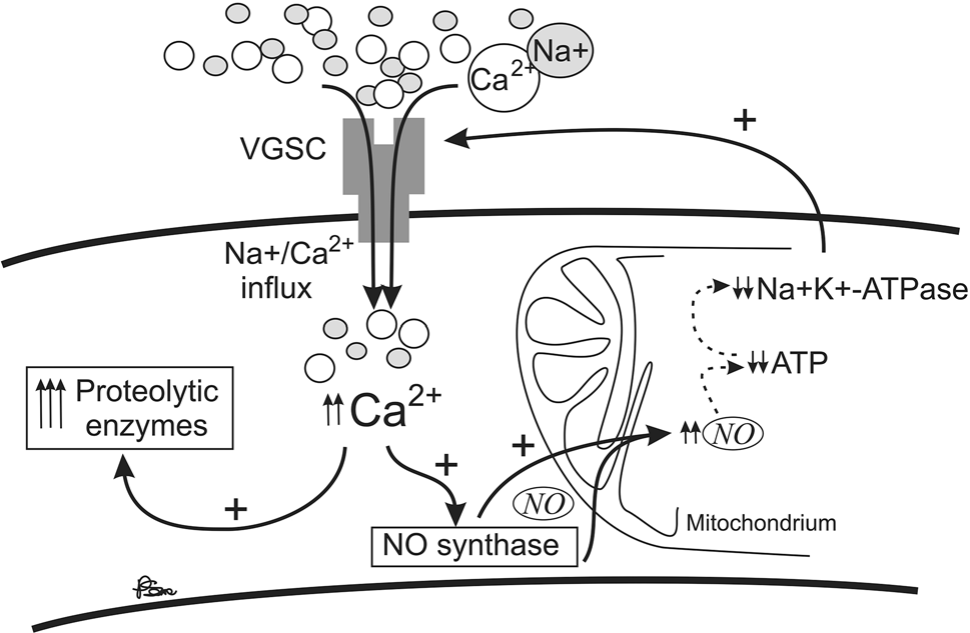
Positive feedback loop of Na/Ca influx to the neuron cell with a potential damaging effect in multiple sclerosis (*VGSC* voltage-gated sodium channel, *NO* nitric oxide, *ATP* adenosine triphosphate, Na^+^K^+^-ATPase sodium–potassium adenosine triphosphatase)

β subunits (β1–β4) combine in vivo with either β1 or β3 through non-covalent bonding and with either β2 or β4 via a covalent bond [101–104]. Numerous studies have revealed the presence of Na_v_1.1, Na_v_1.2, Na_v_1.3 [105], Na_v_1.6 [106], and Na_v_1.5 [107] in rodent astrocytes. Sodium channels in these glial cells are localized to the plasma membrane, where they mediate sodium currents [108]. The star-shaped glial cells situated in the CNS take an essential part in the response of the CNS to injury, including inflammation and degeneration in MS. Herzog et al. have shown that VGSCs can contribute to axonal injury in MS by providing a pathway for sustained sodium influx that drives the Na^+^/Ca^2+^ exchanger to import calcium into axons [109]. Elevated calcium levels can activate nitric oxide synthase and deleterious proteolytic enzymes [109–112]. The harmful effects of nitric oxide on mitochondrial function include a reduction in adenosine triphosphate (ATP) levels and an exhaustion of sodium–potassium adenosine triphosphatase (Na^+^K^+^-ATPase), hence compromising the axons’ capacity to maintain normal transmembrane sodium gradient. This action provides a positive feedback loop that imports even more intracellular calcium, thereby further enhancing the damage [113]. Consequently, these mechanisms lead to axonal injury and further to disability (Fig. 1).

Numerous studies have shown that partial blockade of voltage-gated sodium channels could result in neuroprotection in patients with MS [112]. Indeed, the axonal protection has been demonstrated in animals with experimental autoimmune encephalomyelitis (EAE) by means of the sodium channel blocking drugs flecainide [114, 115], safinamide [115], phenytoin [116], and, recently, lamotrigine [117]. Neuroprotection is emerging as a potentially important strategy for preventing disability progression in MS [118]. In contrast, some clinical studies do not support the protective role of VGSC blockers in MS. Counihan et al. studied 400 patients, 51 of whom received CBZ symptomatic therapy (average duration of therapy was 27 months), and showed that the long term exposure to the VGSC-blocking drug CBZ does not affect the long-term disability and disease progression in MS patients, despite studies in animals suggesting a neuroprotective role of VGSC blockers [119]. Furthermore, using CSF neurofilament (NfH) as a good surrogate marker of neurodegeneration in MS, Gnanapavan et al. revealed no benefit of lamotrigine in the prevention of axonal breakdown by lowering NfH levels compared to the placebo arm [120]. The protective role of lamotrigine is also disputed by Kapoor et al. [121], but it is premature to fully dismiss this hypothesis.

## Sodium chloride intake and MS

High intake of sodium chloride is currently considered to be a potentially important factor influencing the onset of MS. Changes in eating habits that have occurred in recent decades in developed countries may account for an increase in incidence of MS and other autoimmune diseases [122]. Today’s typical Western diet includes more sodium chloride than it was in the past [123]. Therefore, popular processed meals, such as “fast food”, contain approximately 100 times more sodium chloride than homemade meals [124, 125]. Increased hypertonicity can stimulate the immune system [126], and furthermore, superior sodium chloride uptake can affect the innate immune system [127].

**Fig. 2:**
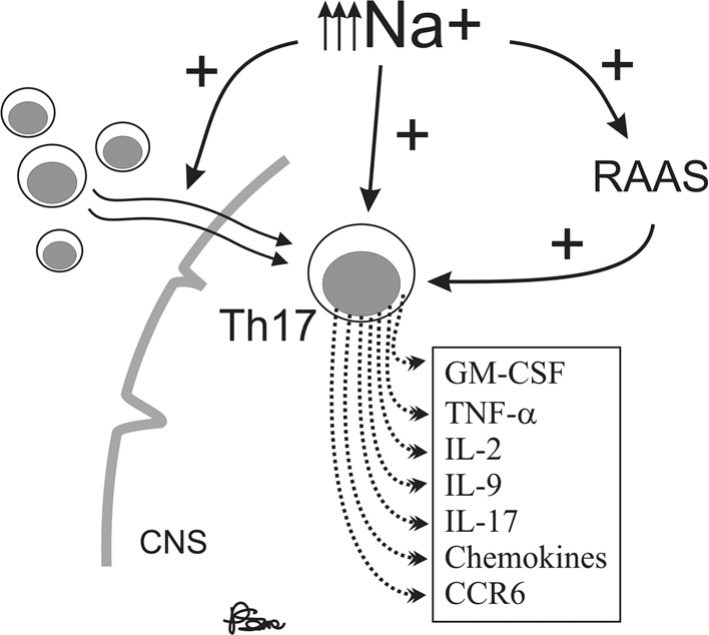
Probable impact of high sodium diet on immune functions in multiple sclerosis patients (*CNS* central nervous system, *RAAS* renin–angiotensin–aldosterone system)

Recent studies have demonstrated the importance of interleukin-17 (IL-17)-induced CD4+ Th17 cell population in autoimmune diseases [128]. Kleinewietfield et al. showed that the addition of a modest amount of NaCl (40 mM) to a culture of differentiating Th17 cells caused a roughly logarithmic augmentation of IL-17A in naïve CD4 cells in vitro and this process was mediated by p38/MAPK, NFAT5, and SGK [122]. Moreover, high-salt concentration results in growth of pathogenic phenotype of Th17 cells [122, 129, 130]. Thus, the change in eating habits that includes a high amount of salt may contribute to the recent increase in MS incidence through the induction of pathogenic Th17 cells [122, 126, 128]. The Th17 cells induced by high-salt concentration upregulate the production of pro-inflammatory cytokines GM-CSF, TNFα, IL-2, IL-9, several chemokines [131, 132], and CCR6 [133], which are essential for the autoimmune function of Th17 cells. Higher Na^+^ concentration, such as that between 160 and 250 mM, in the interstitium and lymphoid tissue and significantly lower concentration of Na^+^ in plasma, approximately 140 mM, are likely to be the mechanism for decreasing the inflammatory response in the blood while favoring immune activation in lymphoid tissues or with migration of cells into tissue [127, 134]. Otherwise, diet rich in salt can affect the severity of the disease. Kleinewietfeld et al. showed that mice fed salty meals developed deterioration of EAE, with an increase in Th17 cell number and augmented infiltration of Th17 cells into the CNS [122]. Moreover, in an observational trial on 122 MS patients, Farez et al. demonstrated that the disease exacerbation rate was 2.75-fold in participants with medium salt intake (2–4.8 g/day) (95% CI 1.3–5.8) and 9.95-fold in participants with high sodium intake (4.8 g/day or more) (95% CI 1.4–11.2) compared with the low-intake group (under 2 g/day) [135]. This finding may be due to the fact that sodium concentration is tightly regulated within narrow limits regardless of large variations in sodium consumption, due to its importance in general metabolism [136]. The renin–angiotensin–aldosterone system (RAAS), which is a major regulator of blood pressure, also significantly affects autoimmunity in many diseases which include MS and its animal model—EAE. Han et al. showed that peptides related to the RAAS are present in CNS lesions of MS patients [137]. Sodium chloride, among many other physiological effects, modulates the renin–angiotensin system [138]. Interestingly, the activation of renin and angiotensin has been implicated in the pathogenesis of EAE [139]. Furthermore, increases in systolic blood pressure similar to those observed with high-salt consumption have recently been shown to be associated with the disruption of white matter integrity in young normotensive individuals [140]. In addition, Platten et al. demonstrated an increase in the expression of angiotensin receptor 1 (AT1R) in lymph node cells, indicating that AT1R is activated in antigen-specific T cells during the peripheral immune response to autoantigens. In addition, angiotensin II (AII) binding was augmented in Periodate–Lysine–Paraformaldehyde (PLP)-activated CD4 + T cells and to a lesser extent in activated CD11b + monocytes. Immunization with PLP139–151 led to an induction of AII in CD4 + T cells, CD11b + monocytes, and to an increase in serum AII levels, demonstrating that the RAAS is activated in peripheral immune cells. Pretreatment of mice immunized with PLP139–151 with the angiotensin converting enzyme (ACE) inhibitor lisinopril {N2-[(S)-1-carboxy-3-phenylpropyl]-L-lysyl-l-proline} or the AT1R antagonist candesartan (3-{[2′-(2 H -tetrazol-5-yl)biphenyl-4-yl]methyl}-2-ethoxy-3 H -benzo[d]imidazole-4-carboxylic acid) resulted in suppression of Th1 and Th17 cytokine release and up-regulation of immunosuppressive cytokines, such as IL-10 and transforming growth factor-β (TGF-β) [141]. Probable impact of high sodium diet on immune functions in MS patients was presented in Fig. 2.

Hucke et al. revealed a multidirectional activity of sodium chloride-rich diet in both humans and mice. Sodium chloride-rich diet promotes CNS autoimmunity, increases macrophage responses, skews the balance towards a pro-inflammatory M1 phenotype in macrophages, alters MAPK signaling in macrophages, and induces a pro-inflammatory phenotype in human monocytes [142]. In addition, Yi et al. demonstrated that high-salt intake promotes an increase in human serum monocytes, which play a pivotal role in the development of various immunological diseases [143]. Furthermore, Jörg et al. showed that a high-salt diet in the early phase of neuroinflammation mainly acts on Th17 cells and is independent of myeloid cells. This finding can help elucidate the impact of a high-salt diet on the emergence and course of autoimmune diseases [144]. Krementsov et al. demonstrated an increase in blood–brain barrier permeability and brain pathology in mice as a consequence of a high-salt diet, but did not demonstrate augmentation of Th17 or Th1 responses. Moreover, this study showed that the effects of dietary sodium on autoimmune neuroinflammation are sex-specific, genetically dependent, and CNS-mediated [145]. Furthermore, Zhou et al. demonstrated that a short-term increase in dietary salt intake could induce the expansion of CD14++CD16+ monocytes, as well as an increase in monocyte platelet aggregates (MPAs), which might be the cellular basis of high-salt-induced end organ inflammation and potential thromboembolic risk, independent of changes in blood pressure [146]. In addition, Hernandez et al. reported that excess dietary sodium intake lowers immunosuppressive actions of human and murine Foxp3+ Tregs in vitro and in vivo and is associated with increased Treg IFNγ secretion in vivo [147]. Data concerning the immunological effects of sodium chloride intake are shown in Table 2.

**Table 2:** Immunological effects of sodium chloride intake

Examined subjects	Time (days)	Sodium intake	Observed effects	
C57BL/6J mice	20	Na^+^-rich diet	Increase in Th17 cells proliferation Exacerbation of EAE	[121]
Human and rabbit PBMC	–	Na^+^ hipertonic medium 25–30 mM (in vitro)	T cell proliferation was doubled in 25 mM medium Increased hypertonicity (>40 mM in human cells; >80 mM in rabbit cells) caused progressive Suppression of proliferation monocyte functions augmentation	[126]
Cd4 Cre Sgk1 fl/fl mice and WT mice	21	High-salt diet	Increase in EAE severity in WT mice, but not in SGK1-deficient mice Increase of Th17 cells in mLN and CNS of WT mice, but not in SGK1-deficient mice Increase in IFN production by T cells in the CNS in WT mice Increase in IL-17 synthesis by CD4+ T cells	[130]
RRMS patients	720	Dietary 2–4.8 g/day of Na^+^ intake	Increase in exacerbation rate (2–4 fold) in patients with medium or high sodium diet Increase in the risk of developing a new MRI lesions in high Na^+^ diet patients	[135]
C57BL/6J mice	50	Na^+^-rich diet	Increase in murine Th17 and Th1 cells Increase in IL-17A and IFN-γ secretion	[144]
C57BL6/J mice and SJL/JCrHsd mice	–	High-salt diet	Exacerbation of disease in M and F of C57BL6/J mice but only in F of SJL/JCrHsd mice No influence on C57BL6/J mice carrying a 129/Sv-derived interval on chromosome 17 No influence on Th17 or Th1 cells Increase in blood–brain barrier permeability and brain pathologies	[145]
*Foxp3*-GFP reporter mice	21	Na^+^-rich diet	Induction of Th1-type phenotype Impairement in Treg function (IFNγ-dependent)	[147]
Healthy human	205	Dietary NaCl reduction	Decrease in monocytes counts Decrease in IL-6 (30%), IL-23 (90%) and IL-17 concentration Increase in IL-10 level (threefold) Slightly decrease in VEGF-C serum concentration	
Healthy human	17	High-to-low NaCl diet	Increase in CD14++ and CD16+ monocytes proliferation Increase in intracellular ROS production	

*EAE* experimental autoimmune encephalomyelitis, *PBMC* peripheral blood mononuclear cells, *ROS* reactive oxygen species

In conclusion, recent reports have demonstrated a potential pro-inflammatory role of excess sodium chloride intake in the pathogenesis of autoimmune and neurodegenerative diseases, both in vitro and in vivo, although the outcomes of these studies are not unanimous. Nevertheless, the current knowledge suggests that a low-salt diet (<5 g/day) might be beneficial in the prevention and treatment of autoimmune diseases, including MS.
